# Clinical and Radiological Outcomes of Patients With Anterior Acetabulum Fractures Treated by the Modified Stoppa Approach

**DOI:** 10.7759/cureus.49237

**Published:** 2023-11-22

**Authors:** Rakesh Kumar, Anand Shankar, Ashutosh Kumar, Rishabh Kumar

**Affiliations:** 1 Trauma and Emergency, Indira Gandhi Institute of Medical Sciences, Patna, IND

**Keywords:** pelvis acetabulum, acetabulum fractures, pelvic acetabulum fractures, pelvi-acetabulum, matta criteria, transverse acetabular fracture, acetabular fracture, modified stoppa approach

## Abstract

Introduction

Acetabular fractures are intra-articular fractures involving the lower extremity's weight-bearing dome. These fractures require an anatomical reduction of the fracture fragments. This aim can be accomplished by the selection of an appropriate surgical approach. This study aimed to analyze the clinical and radiological outcomes of patients with fractures in the anterior part of the acetabulum who were treated by the modified Stoppa approach.

Methods

This prospective observational study was conducted from April 2022 to September 2023. The inclusion criteria were: (i) age between 18 and 70 years, (ii) displaced acetabular fracture (displacement > 3 mm), (iii) within three weeks of trauma (iv) acetabular fractures with involvement of anterior column. Exclusion criteria included: (i) patients with visceral injuries requiring colostomy, (ii) pathological fracture, (iii) open fractures of the acetabulum, and (iv) neglected fracture (more than three weeks). Intraoperative data regarding surgical time, amount of blood loss, and incidence of intraoperative complications were recorded. In the postoperative period, anteroposterior X-ray and Judet views of the pelvis X-ray were obtained. Matta criteria were used to judge the quality of Fracture reduction and fixation. All the patients to be included in this study had undergone a minimum follow-up duration of six months. At the last follow-up, an assessment of the functional outcome of the affected hip by Merle d’Aubigné Hip Score and Harris Hip Score was done.

Results

Twenty-four patients were included in the study. The mean patient age was 36.08±11.65 years. Eighteen patients were male (75%) and six patients were female in this study. All acetabular fractures were due to high-energy trauma: road traffic accidents in 22 cases (91%) and fall from height in two cases (9%). According to Judet & Letournel's classification, there were 13 T-type fractures, five transverse fractures, and six associated both column fractures. The mean duration of surgery was 152.08 ±29.19 minutes, and the mean intraoperative blood loss was 277.08±85.95 ml. Intraoperatively one unit of blood transfusion was done in most cases. There were intraoperative complications of rent in the external iliac vein in two patients. Postoperative X-rays showed anatomical reduction in 17 cases, imperfect reduction in five cases, and poor reduction in two cases. Functional outcome of the hip by Merle d’Aubigné Hip Score was very good in 15, good in four, fair in three, and poor in two patients. Similar functional outcomes were obtained with the Harris Hip Score.

Conclusion

The results of the current study demonstrated that the modified Stoppa approach allows good visualization of the pelvic brim, quadrilateral surface, and posterior column. Lesser experienced orthopedic surgeons should utilize this approach to get good radiological and functional outcomes.

## Introduction

Acetabular fractures are intra-articular fractures that involve the weight-bearing dome of the lower extremity. Their close proximity to neurovascular structures and internal organs makes them complex injuries. The optimal outcome in cases of acetabular fractures needs anatomic reduction of fracture fragments with stable column fixation. Surgical exposure to acetabulum has a steep learning curve and thus, surgical outcomes are better in experienced hands. Acetabulum has been traditionally approached through the ilioinguinal approach originally described by Letournel for fractures in the anterior part and the Kocher-Langenbeck (KL) approach for fractures in the posterior part [1]. The ilioinguinal approach needs extensive dissection of the neurovascular bundles in and around the femoral canal, which increases surgical time and intraoperative blood loss. The combined view obtained through the three windows in the ilioinguinal approach offers only a limited view of the quadrilateral plate and thus limited assessment of the fracture fragments. Only indirect reduction maneuvers can be utilized for fracture reduction and fixation.

Rives et al. [2] and Stoppa et al. [3] initially employed the classic Stoppa approach in inguinal hernia surgery. Cole and Bolhofner [4] and Hirvensalo et al. [5] modified the Stoppa approach and used it to approach the anterior acetabulum and pelvic bone. The Stoppa approach offers many distinct advantages: it provides a sufficient area of visualization for pelvic ring exposure, does not involve exposure of the femoral canal or iliac vessels, and provides sufficient reduction in anterior wall and column fractures. It also allows adequate reduction of the articular fragment impacted into the weight-bearing dome [6]. This approach is relatively easier than the Ilioinguinal approach, decreases surgical time, and is replicable by less experienced surgeons. This study aimed to analyze the clinical and radiological outcomes of patients with fractures in the anterior part of the acetabulum who were treated by the stoppa approach.

## Materials and methods

This prospective observational study was conducted from April 2022 to September 2023 at a tertiary-level institution, Indira Gandhi Institute of Medical Sciences, Patna, in Eastern India. The study was carried out after obtaining ethical clearance from the Institutional Ethics Committee at Indira Gandhi Institute of Medical Sciences, Patna (474/IEC/IGIMS/2022). The inclusion criteria included: (i) age between 18 and 70 years, (ii) displaced acetabular fracture (displacement > 3 mm), (iii) within three weeks of trauma and (iv) acetabular fractures with involvement of anterior column. Exclusion criteria included: (i) patients with visceral injuries requiring colostomy, (ii) pathological fracture, (iii) open fractures of the acetabulum, and (iv) neglected fracture (more than three weeks).

Preoperative workup: Patients were assessed at the time of presentation to the hospital. Detailed histories were taken with the recording of demographic variables, mode of injury, and associated medical illness. An in-depth, clinical assessment was carried out to rule out life-threatening injuries. Adequate analgesia was provided along with traction of the affected limb. Radiological investigations of X-rays and CT scans with 3D reconstruction films were done when the condition of the patient permitted.

Surgical technique: All the patients were administered second-generation cephalosporin 30 minutes before skin incision. The patients were positioned in the supine position and administered spinal/general anaesthesia. A radiolucent pillow was placed below the knee joint and the leg longitudinally to keep the hip flexed. The lower abdomen along with the involved lower limb (up to mid-thigh) was prepared and draped. The surgeon stood on the opposite side of the fracture. A 10 cm transverse incision was made about 2 cm proximal to the pubic symphysis. The subcutaneous layer was incised in line with the skin incision. The deeper layer was separated bluntly. The shiny aponeurotic fibers of external oblique muscles on both sides were identified and traced to the linea alba in the midline. The linea alba was incised in the midline from pubic symphysis below towards the umbilicus above. The retropubic space of retzius was bluntly dissected with fingers and the urinary bladder was retracted posteriorly. The insertion of the ipsilateral rectus abdominis muscle was elevated off the ipsilateral superior pubic ramus, anterosuperior aspect of the pubic body, and pubic tubercle by diathermy. The rectus abdominis was retracted laterally. The neurovascular bundle remained inside the retracted mass. If corona mortis was encountered, it was ligated or cauterized depending upon its caliber. The iliopectineal fascia was incised to expose the pelvic brim and the obturator fascia was incised to expose the quadrilateral plate and the posterior column of the acetabulum.

Fracture reduction and fixation technique: To bring the femoral head smoothly out of the pelvic cavity, a reduction maneuver in the form of hip flexion, internal rotation, and pulling out the femur was done. This was assisted by pushing the fracture fragments by a ball spike pusher from inside the pelvic cavity. In some cases, a 5.5 mm Schanz screw was inserted through the trochanter into the femoral neck. A universal T handle was then attached to the Schanz screw and was to apply lateral traction. After adequate reduction was confirmed in intraoperative C arm pictures, fixation with pelvic reconstruction plates was done. All the implants were fixed under direct vision and checked for being extra-articular by rotation of the hip and fluoroscopy. After the anatomical reduction of the anterior column, the femoral head was concentrically reduced under the lateral articular dome.

Intraoperative data regarding surgical time, amount of blood loss, and incidence of intraoperative complications were recorded. In the postoperative period, anteroposterior X-ray and Judet views of the pelvis X-ray were obtained. Matta criteria were used to judge the quality of fracture reduction and fixation: 0-1 mm displacement was regarded as anatomical, 2-3 mm as imperfect, and >3 mm displacement was considered poor in X-ray film. As per the patients’ conditions, early range of motion and non-weight-bearing exercises of the affected limb were conducted for six weeks. Patients were followed up after six weeks, three months, four-and-a-half months, and six months and one year. Partial weight-bearing exercises were started after 12 weeks and full weight-bearing was individualized.

The endpoint of the follow-up period in this study was 30 September 2023. All the patients to be included in this study had undergone a minimum follow-up duration of six months till that time. At this time, we assessed the functional outcome of the affected hip by Merle d’Aubigné Hip Score and Harris Hip Score. Merle d’Aubigné Hip Score is based on a 7-point scale of pain, mobility, and ability to walk with each having a minimum value of 0 and a maximum value of 6. The summation of scores for pain and mobility results in an absolute estimation of hip function: Very good (11-12), Good (10), Medium (9), Fair (8), and Poor (≤7). Harris's hip score is categorized as Excellent (≥90), Good (80-89), Fair (70-79), and Poor (<70).

All statistical analyses were done using SPSS software, version 20.0 (IBM Corp., Armonk, NY). Categorical data are presented as absolute numbers & percentages (%), and noncategorical/numerical as mean and SD (standard deviation). Independent t tests were performed for comparing noncategorical variables. The level of statistical significance was set at p-value < 0.05.

## Results

Demographic characteristics of patients: 24 patients were included in the study. The mean patient age was 36.08±11.65 years. Eighteen patients were male (75%) and six patients were female in this study (male/female=3/1) (Table 1). Among the 24 acetabular fractures, the right side was involved in 14 fractures and the left side was involved in 10 fractures. All acetabular fractures were due to high-energy trauma: road traffic accidents in 22 cases (91%) and fall from height in two cases (9%). According to Judet and Letournel's classification, there were 13 T-type fractures, five transverse fractures, and six associated both column fractures. There were additional injuries: other injuries in the pelvis in six patients, ipsilateral patella fracture in one patient, a fracture of the distal end of the radius in one patient, and a fracture of the shaft of the tibia in the ipsilateral limb in one patient. The details of the patients are given in Table 1.

**Table 1:** Patient's demographic characteristics Data is shown as Mean±SD, N (%) SD: Standard Deviation

Variable	Value (percent)
Age (years)	36.08 ± 11.65
Gender	
Male	18 (75%
Female	6 (25%)
Side of Injury	
Right Side	14 (58%)
Left Side	10 (42%)
Mechanism of Injury	
Road traffic accident	22 (92%)
Fall from height	2 (8%)
Additional Injuries	
Other injuries in the pelvis	6 (25%)
Ipsilateral patella fracture	1 (4%)
Contralateral distal end of radius fracture	1 (4%)
Ipsilateral shaft of tibia fracture	1 (4%)
Fracture classification	
Associated both column	6 (25%)
Transverse	5 (21%)
T-Type	13 (54%)
Operative time (minutes)	152.08 ± 29.19
Blood loss (ml)	277.08 ± 85.95

The average duration of surgery was 150 minutes (range 120 to 210 minutes). The mean intraoperative blood loss was 250 ml. Intraoperatively one unit of blood transfusion was done in most cases. There were intraoperative complications of rent in the external iliac vein in two patients. Both were managed with suturing of the rent in the same sitting. In both cases, three units of blood were transfused intraoperatively. Two patients had developed superficial skin infections, which were treated by wound debridement and appropriate antibiotics.

Postoperative X-rays showed anatomical reduction in 17 cases, imperfect reduction in five cases, and poor reduction in two cases. Functional outcome of the hip by Merle d’Aubigné Hip Score was very good in 15, good in four, fair in three, and poor in two patients. Similar results were obtained in the same patients with the Harris Hip Score. Those two patients had early signs of avascular necrosis of the femoral head in the latest X-rays. Table 2 describes the outcomes of the patients.

**Table 2:** Patient's radiological and clinical outcomes Data is shown as N (%)

Classification		Criteria	No. of Patients (percent)	
Quality of Fracture Reduction (according to Matta Criteria) on X ray				
Anatomical		0-1 mm displacement	17 (71%)	
Imperfect		2-3 mm displacement	5 (21%)	
Poor		>3 mm displacement	2 (8%)	
Merle d’Aubigné Hip Score				
Very good	11-12			15 (63%)
Good	10			4 (17%)
Medium	9			0
Fair	8			3 (12%)
Poor	≤7			2 (8%)
Harris Hip Score				
Excellent	(≥90)			15 (63%)
Good	(80-89)			4 (17%)
Fair	(70-79)			3 (12%)
Poor	(<70)			2 (8%)

**Figure 1:**
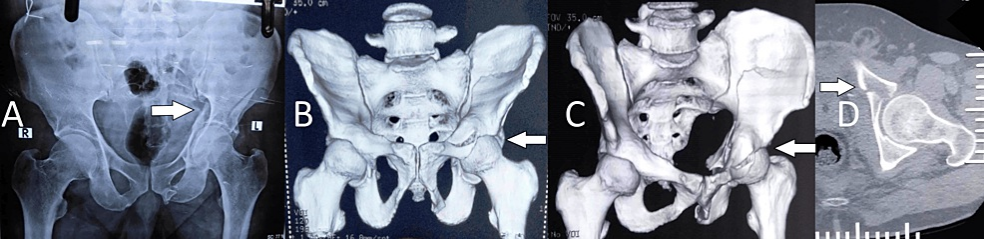
Preoperative radiography and computed tomography images A) Pelvis anterior-posterior (AP) radiograph; B) Anterior-posterior view in three-dimensional reconstruction of computed tomograph (CT); C) Iliac oblique view in three-dimensional reconstruction; D) Axial view in CT scan.

**Figure 2:**
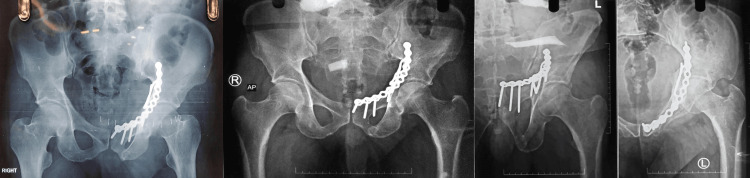
Postoperative radiographs A) Postoperative pelvis anterior-posterior (AP) radiograph; B) Follow-up pelvis anterior-posterior radiograph at six months C) Follow-up pelvis outlet view radiograph at six months D) Follow-up pelvis inlet view radiograph at six months.

**Figure 3:**
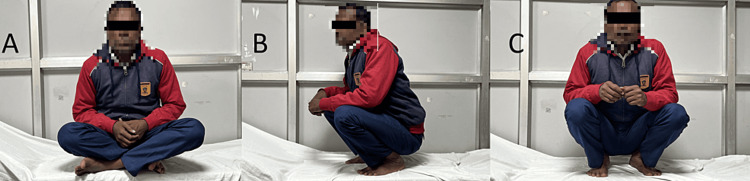
Follow up Clinical photograph of the patient A) Sitting cross-legged B) Squatting turned to right C) Squatting

Figures 1-3 illustrate one of the case done at our institution.

## Discussion

In developing countries like ours, the burden of acetabular fractures is significant. Most of these fractures are due to high-energy injuries, such as road traffic accidents. The mean age of patients in this study was 36.08 years. Males constituted 75% of patients in this study. This was mainly due to the age structure in this country, where young males constitute the major working population. This predominance of males and young patients with the most common mode of injury as road traffic accidents was also reported by other authors in this country [7,8]. Further there is a severe deficit of appropriately trained acetabular surgeons in developing countries [8]. This affects the outcome of such fractures as many orthopaedic surgeons treat these fractures conservatively or there is a delay in referral to tertiary centres which hampers functional outcome. As the hip joint is a major weight-bearing joint of the body, this fracture requires an absolute anatomical reduction of the fracture fragments [9,10]. This anatomical reduction can only be achieved by utilizing an appropriate surgical approach. The ilioinguinal approach is the gold standard for the surgical treatment of anteriorly situated acetabular fractures. In recent years, the modified Stoppa approach has gained popularity due to less extensive dissection, less surgical time, and direct visualization of the inside of the pelvis and quadrilateral plate. However, both the ilioinguinal and modified Stoppa approaches do not offer direct visualization of the articular surface. The learning curve of the Stoppa approach is less steep than the ilioinguinal approach and gives good outcomes. 

As the surgeon peeps into the pelvic cavity by standing on the opposite side of the fracture, it is imperative to have good retraction of muscles and soft tissues. The ipsilateral rectus muscle needs to be elevated from the anterosuperior surface of the pubic body. This increases the retractable length of the rectus muscle. We keep the operative hip in a flexed position by keeping a pillow longitudinally beneath the knee and leg. This relaxes the iliopsoas muscle, which must be retracted laterally in the deeper planes. The external iliac vessels also get retracted along with the iliopsoas muscle. With appropriate retraction, the whole surface of the pelvic brim, from the pubic symphysis to the iliac buttress along with the sacroiliac joint, is visible to the surgeon. Corona mortis was lacerated in most cases by the fracture fragments in the vicinity of its location. In cases where it was present, we preferred to ligate it. It is better to tie the corona mortis as during subperiosteal dissection of the pelvic brim, there is traction on the larger vessels that the corona mortis connects, namely the obturator artery and the iliac artery. In this case, the bleeding can be massive and difficult to control [11]. Care was taken to protect the obturator nerve and vessels throughout the surgery. In our initial cases, we experienced increased surgical time and complications. There was a laceration of the external iliac vein in two cases which increased the surgical time and amount of intraoperative blood loss and blood transfusion. These lacerations were managed by suturing of the rent in the external iliac vein. The hip was not flexed in these cases leading to less retraction of the iliopsoas muscle. However, after we started flexing the position of the hip, these complications did not occur.

T-type fractures, transverse fractures, and associated both column fractures constituted the cases in this study. This was due to the inclusion criteria of this study. The Stoppa approach is very useful in transverse and T-type fractures, as the fracture line in the posterior column is high and closer to the sciatic notch in these cases. For the fixation of the posterior column, we utilized the KL approach in all the cases. Radiological assessment was done by postoperative X-rays, AP views and Judet views. Postoperative CT scan was not utilized routinely due to their cost and radiation hazards although they are more sensitive regarding reduction quality. Congruent joint obtained after anatomical reduction led to good functional outcomes. This congruency of the acetabular surface and femoral head is required more in the zone of the weight-bearing dome as weight is transferred through that zone. Functional outcome in this study was measured by Merle d’Aubigné Hip Score and Harris hip score. These scores have high inter-observer reliability [12].

Many authors have published a large series of patients operated by the modified Stoppa approach. Hirvensalo et al. used the modified Stoppa approach in 164 cases of pelvic and acetabular surgery. In that series, 138 patients (84.1%) had an anatomic reduction, 9% were graded as fair, and 7% were graded as poor. They found that 80% of patients had a Harris hip score of 75 or greater on clinical examination and functional outcome scoring [5]. Sagi et al. described 50 cases, of which 92% had an excellent or good reduction of acetabular fractures [13]. However, the use of a lateral window was required in 60% of the cases. Anderson et al. reported an 82% anatomic reduction rate, 18% imperfect and 0% poor reduction in postoperative radiographs in a consecutive cohort of 17 patients [14]. In this study, we achieved anatomical reduction in 17 (71%) cases, imperfect reduction in five (21%) cases, and poor reduction in two (8%) cases. The results of this study have been compared with other recent studies in Table 3.

**Table 3:** Comparison of quality of fracture reduction and functional outcome of this study with recent studies Data has been presented as N (%); *- Functional outcome was analyzed by Majeed Score; **- Functional outcome was analyzed by Harris Hip Score.

Authors	Year of Study	Duration of Study	Study Design	No. of Patients	Quality of Fracture Reduction (Matta Criteria)			Functional Outcome (Merle D' Aubigne and Postel Score)			
					ANATOMICAL	IMPERFECT	P00R	EXCELLENT	GOOD	FAIR	POOR
This study	2023	April 2022 to September 2023	Prospective cohort study	24	17 (71%)	5 (21%)	2 (8%)	15 (63%)	4 (17%)	3 (12%)	2 (8%)
Yang et al. [15]	2022	January 2009 to January 2016	Retrospective study	57	23 (40%)	22 (39%)	12 (21%)	17 (30%)	25 (44%)	4 (7%)	11 (19%)
Kwak et al. [16]	2022	June 2018 and June 2020	Retrospective study	16	14 (88%)	1 (6%)	1 (6%)	4 (25%)	9 (56%)	3 (19%)	0
Ciolli et al. [17]	2021	February 2018 and February 2020	Retrospective study	34	26 (76%)	7 (21%)	1 (3%)	6 (18%)	16 (47%)	10 (29%)	2 (6%)
Chen et al. [18]	2021	January 2015 and June 2017	Retrospective cohort study	50	22 (44%)	21 (42%)	7 (14%)	18* (36%)	20* (40%)	11* (22%)	1* (2%)
Yang et al. [19]	2020	January 2014 to January 2018	Retrospective comparative cohort study	32	21 (66%)	7 (22%)	4 (12%)	15 (47%)	7 (22%)	6 (19%)	4 (12%)
Yang et al. [20]	2020	January 2015 and December 2017	Retrospective cohort study	18	12 (67%)	5 (28%)	1 (5%)	11 (61%)	4 (22%)	2 (11%)	1 (6%)
Singh et al. [21]	2020	October 2017 to April 2019	Prospective cohort study	30	26 (87%)	3 (10%)	1 (3%)	13 (43%)	15 (50%)	2 (7%)	0
Nayak et al. [22]	2020	February 2017 to October 2018	Prospective cohort study	23	18 (78%)	2 (9%)	3 (13%)	6 (26%)	13 (56%)	2 (9%)	2 (9%)
Al Adawy et al. [23]	2020	June 2015 to May 2019	Randomized Control Trial	20	15 (75%)	3 (15%)	2 (10%)	11 (55%)	7 (35%)	0	2 (10%)
Kilinc et al. [24]	2019	February 2013 and June 2016	Retrospective study	57 (6 patients had bilateral fractures)	52 (83%)	9 (14%)	2 (3%)	27** (43%)	23** (36%)	4** (6%)	3** (5%)

Avascular necrosis (AVN) has been reported at a level of 5.6% in patients undergoing surgery for a fracture of the acetabulum and is more common in subjects with traumatic hip dislocation [25]. Early evidence of AVN was seen in two (8%) of the 24 acetabular fractures in this study. Functional outcomes in these two patients were poor. Tannast et al. reported significant associations of secondary osteoarthritis with patients aged > 40 years, postoperative incongruence of the acetabular roof, involvement of the posterior acetabular wall, acetabular impaction, a femoral head cartilage lesion, initial displacement of the articular surface of >20 mm, and use of the extended iliofemoral approach [10].

This study had some limitations. First, it had a small sample which prevented us from drawing statistically significant conclusions. Secondly, the functional outcomes of the patients were considered at six months. The functional results can change in the long term. So Late complications can also occur. Thirdly, with increasing surgical experience, better results were obtained in later patients as compared to initial ones. Though we achieved good functional results, longer follow-ups, and a larger study population would have further strengthened our findings.

Bones of the pelvis and acetabulum are curved bones, and thus implants are also curved. In the initial years of orthopaedic surgeons they mainly do surgeries of long bones of extremities whose implants are almost straight. Pelvi-acetabular surgery demands a transition from straight bones to curved bones. There is a need for a relatively easy surgical approach with a less steep curve to encourage lesser experienced orthopaedic surgeons. Further prospective studies should be conducted on the modified Stoppa approach with a larger sample size and long duration of follow up.

## Conclusions

The results of the current study demonstrated that the modified Stoppa approach allows good visualization of the pelvic brim, quadrilateral surface, and posterior column. Less experienced orthopedic surgeons should utilize this approach to get good radiological and functional outcomes. However, for the best patient outcomes, surgeons should be familiar with both the ilioinguinal approach and the modified Stoppa approach. As this study had a small sample size and short follow-up, further research with a larger sample size and longer follow-up is needed to fully validate the findings of this study.
